# Early-life antibiotic exposure increases the risk of childhood overweight and obesity in relation to dysbiosis of gut microbiota: a birth cohort study

**DOI:** 10.1186/s12941-022-00535-1

**Published:** 2022-11-03

**Authors:** Ping Li, Xuelian Chang, Xiaoyu Chen, Chuan Wang, Yu Shang, Dongyi Zheng, Kemin Qi

**Affiliations:** 1 Laboratory of Nutrition and Development, Key Laboratory of Major Diseases in Children’s Ministry of Education, Beijing Pediatric Research Institution, Beijing Children’s Hospital, Capital Medical University, National Center for Children’s Health, No.56 Nan-li-shi Road, 100045 Beijing, China; 2 Department of Child Health Care, Chaoyang District Maternal and Child Health Care Hospital, 100021 Beijing, China; 3 Department of Child Health Care, The First Hospital of Tsinghua University, No. 6. Jiu-xian-qiao 1st Street, 100016 Beijing, China

**Keywords:** Early-life, Antibiotic exposure, Cohort, Obesity, Gut microbiota

## Abstract

**Background:**

Early-life antibiotic exposure is associated with the development of later obesity through the disruption of gut microbiota in the animal models. However, the related epidemiological evidence is still conflicting.

**Methods:**

A birth cohort was consisted of 2140 mother-infant pairs in Chaoyang District Maternal and Child Health Care Hospital in this study. Here, their available antibiotic exposure during the first one year of life was ascertained using a open-ended questionnaire and related anthropometric parameters from the health screening program. The compositions of gut microbiota were comprehensively analyzed by16S rRNA high throughput sequencing. Then the spearman correlations were performed by the multiple covariance-adjusted regressions between the antibiotic exposure with anthropometric parameters and compositions of gut microbiota.

**Results:**

Among the 2140 subjects, the antibiotic exposure during the first one year of life was 53.04%, mainly by Cephalosporins (53.39%) and Erythromycins(27.67%) for the treatment of respiratory tract infection (79.56%), which were not significantly different among the subgroups. Compared to the control group, both childhood overweight and obesity at two and a half years were higher in the antibiotic exposed group, with higher percents of *Faecalibacterium*, *Agathobacter* and *Klebsiella*, and lower percentage of *Bifidobacterium*. Moreover, there were positively potential associations between early-life antibiotic exposure with the accelerated anthropometric parameters and disruption of *Faecalibacterium*, *Agathobacter*, *Klebsiella* and *Bifidobacterium* at two and a half years.

**Conclusion:**

These above results proved that early-life antibiotic exposure was positively associated with the accelerated childhood overweight and obesity from one year to two and a half years by impacting the disorders of *Faecalibacterium, Agathobacter, Klebsiella* and *Bifidobacterium*, which would propose the theoretical basis for rationalizing the personalized antibiotic exposure among the infants to truly reflect the fairness of public health.

**Supplementary information:**

The online version contains supplementary material available at 10.1186/s12941-022-00535-1.

## Background

Obesity is a major challenge of worldwide public health nowadays [1, 2]. Currently, it is estimated that around 124 million children are classified as obese based on the World Health Organization body mass index (BMI) cutoffs [3]. Both overweight and obesity are difficult to treat and tend to track into a broad modifiable spectrum of long-term adverse health consequences such as type 2 diabetes mellitus, hyperlipidemia, hypertension and so on in the childhood, and even adulthood [4]. Although the excessive energy intake and physical inactivity are recognized as the important causes of childhood obesity, but emerging literature has indicated that a wider range of environmental exposure is increasingly suspected to impact the development of later obesity [5, 6]. Hence, identifying the modifiable determinants of overweight and obesity is the pressing research needs. Recently, many evidence have suggested that the abnormal colonization of gut microbiota is preceded as the clinical manifestation of childhood metabolic disorders such as the overweight and obesity [7–9], so given the modifiable mature of gut microbiota could modulate the host metabolism by shifting the infant dietary and environmental elements[1, 10, 11]. Since the first year of life is a critical period for the colonization and maturation of infant gut microbiota with downstream metabolic consequences during this window, which could improve the status of childhood overweight and obesity[12–14].

Recently, there are many increasing epidemiological evidence that could disrupt the early-life microbiome on the progression of obesity [15, 16]. In this critical period, the transmission and maturation of gut microbiota is particularly vulnerable to the external perturbations, especially the antibiotics [9, 17]. Thus, the abuse antibiotic exposure can plausibly impact the host metabolism and compromise the childhood health by affecting the compositions of gut microbiota [18]. Historically, the antibiotics had been used as the growth-promoting agents in the animal aquaculture, which has been raised for the market consumption to increase the adiposity [19–21]. Meanwhile, the linkage correlations between early-life antibiotic exposure and childhood obesity had been convincingly demonstrated in the mice studies, with the weaker associations available from the epidemiological studies [22], in which both a number of cohorts and meta-analysis had proved that early-life antibiotic exposure could increase the risk of childhood overweight and later obesity [23–28]. Despite the other mounting evidence conversely had the inconsistent roles on the childhood obesity likely due to the methodological differences such as the different age, sex, and details of antibiotics (timing, number of courses, class and treatment of disease) [27, 29–31], so it is very important to explore the roles of ealy-life antibiotic exposure in the first year of life on the later overweight and obesity using the cohort with much large samples.

Considering that antibiotic exposure is frequently prescribed among the Chinese infants, a precise assessment of the relationship between antibiotic exposure and childhood overweight and obesity is still important [32]. So a birth cohort including 2140 mother-infant pairs in Chaoyang District Maternal and Child Health Care Hospital from May 2017 to October 2018 was used to determine whether antibiotic exposure during the first one-year of life was independently associated with the development of childhood overweight and obesity to age two and a half years. Then Given the prior evidence was particularly evaluated the differential impacts on the colonization of gut microbiota, which might constitute the theoretical basis for rationalizing the personalized antibiotic exposure among the infants to truly reflect the fairness of public health. It will not only ensure the reasonable utilization of early-life antibiotic exposure in the first one year of life, but also provide a new idea for the prevention of later childhood overweight and obesity.

## Materials and methods

### Study design and participants

We conducted a longitudinal prospective birth cohort, with recruiting 2140 mother-infant pairs in Chaoyang District Maternal and Child Health Care Hospital from May 2017 to October 2018 according to the following strict inclusion and exclusion criteria. Specifically, all subjects were healthy at 20–45 years old without smoking and drinking history at the 6–12 gestational weeks among the Chinese Han population and intended to deliver in this hospital. Meanwhile, the infants should be full-term healthy newborns, exclusive breast-feeding to six months and routinely examined their anthropometric parameters by flowing up to two and a half years old. Conversely, the subjects were excluded if they had the following medical diseases (serious intestinal diseases, hypertension, hypothyroidism, diabetes, heart, liver, kidney and blood system related diseases), long-term history of medicines and chemical reagents (hormone, formaldehyde and thyroxine related drugs) during the whole pregnancy. Moreover, the subjects with maternal antibiotic exposure during the whole breastfeeding and their infants with birth defects, genetic and metabolic diseases, ischemia, hypoxia, premature and low body weight at birth were also excluded. Furthermore, the subjects could be actively withdrew from this study if they were loss of interests and follow-up records, and the other unknown reasons.

This clinical research was approved by the Ethics Committee of Beijing Children’s Hospital, Capital Medical University (No: 2016-20), which was also recorded at the website of http://www.chictr.org.cn/showproj.aspx?proj=4673 (No: ChiCTR-OCH-14,004,900). And all subjects were granted both the written consents and health insurance portability and accountability act authorization after they were clearly informed the significance of this survey by the trained investigators.

### Basic information questionnaires

The basic maternal characteristics, including the age, height, pre-pregnancy weight, pre-pregnancy overweight and obesity, prenatal weight, education, and medical history and antibiotic exposure during the pregnancy were obtained by the trained investigators through face to face method when the participants were involved in this study. Meanwhile, the infant basic questionnaire such as sex, gestational age, mode of delivery, breast-feeding duration and introduction to solid foods was obtained to get the information when they had their physical examination.

### Biological sample collection

All biological samples were executed in the morning by the trained professional researchers under the strict aseptic conditions and uniform protocols. Exactly, 5mL breast milk was collected at the 3 months physical examination of their infants. Then approximately 1 g fecal samples were obtained using the sterile plastic spoons at two and a half years old. All biological samples were immediately transported to the laboratory and preserved at − 80 °C until use.

### Antibiotic exposure in the first year of life

The antibiotic exposure in the first year of life was drawn by a open-ended questionnaire and patient-recorded medications from the primary care visits at their physical examination of 3 months, 6 months and one year old, in which both the antibiotic exposure and related diseases were recorded as the categorical medication rather than as specific dose (documented as “yes” or “no”) because the specific doses were not always presented. Then all subjects were divided into two groups as the control and antibiotic exposed groups according to the recurrent antibiotic exposure in the first year of life, in which the subgroups (less than 3 months, 3 to 6 months, and 6 months to one year) were respectively obtained as the age of firstly antibiotic exposure.

### Growth and development outcomes from birth to two and a half years old: follow-up

The anthropometric parameters (length and weight) at birth and each subsequent follow-up visits were obtained by the pediatricians using the nearest millimeter (HW-1000HW-2000, China) on a digital measuring board with three repeated measurements to calculate the means, which were then recorded and gotten from the Child Welfare Clinic and School Health-care in detail. The related BMI (kg/m^2^), length for age Z score (LAZ), weight for age Z score (WAZ) and BMI for Z score (BMI Z) were calculated according to the growth curves under 5 years of age using the ANTHRO 2005 software and downloaded from WHO website (http://www.who.int/childgrowth/software/en/).

### Enrolling the subjects to determine the development of gut microbiota

60 subjects were randomly enrolled in the control (n = 1005) and antibiotic exposed groups (n = 1135) to determine the colonization of gut microbiota, whose milk microbiota was also evaluated as the gut microbiota.

### DNA preparation and 16 S rRNA high-throughput sequencing

Within the strictly controlled workplace, total DNA samples in the faeces (approximately 200 mg) and milk among the above enrolling subjects (n = 60/group) were extracted using the QIAamp DNA Mini Kits (Qiagen, Valencia, CA). The isolated DNA samples were eluded in 50µL distilled water with their well controlled quality and quantity.

The variable regions 3 and 4 (V3-V4) in the 16 S rRNA gene were sequenced on the IlluminaHiSeq Platform 2500 using the modified 515 F (5′-GTGCCAGCMG CCGCGGTAA-3′) and 806R (5′-GGACTAC NNGGGTATCTAAT-3′) primers at Novogene (Beijing, China). Then the colonization of gut and milk microbiota was evaluated and analyzed using the NovoMagic platform in Beijing SinoGenoMax Center Co., Ltd (https://magic.novogene.com/customer/main#/login). Exactly, α indicators evaluating the richness (Ace and Chao) and diversity of microbial community (Shannon and Simpson) were calculated using the Faith phylo-genetic diversity. Principal coordinate analysis (PCA) and Non-Metric Multi-Dimensional Scaling (NMDS) analysis based on Bray-Curtis distance as the β diversity indicators were performed to compare the compositions of gut and milk microbiota at the control and antibiotic exposed groups. Through the Lefse, network, ternary plot and evolutionary_tree analysis, the biomarkers with statistical differences were found between all groups, then *t* test and ANOVA (q test) was analyzed the significant differences at the phylum and genus levels. Meanwhile, the contribution differences were quantified by the simper analysis. The Phylogenetic Investigation of Communities by Reconstruction of Unobserved States (PICRUSt) was chosen to predict the meta-genome function. The spearman rank correlations and CCA/RDA analysis were used to measure the associations between the antibiotic exposure with anthropometric parameters and compositions of gut and milk microbiota.

### Statistical analysis

The descriptive statistics for maternal and infant characteristics were included the comparisons of demographic and clinical parameters by SPSS 21.0, in which *P* < 0.05 was recognized as the significant importance.

The normal distribution of outcome variables was evaluated by the Kolmogorov-Smirnov test. All data was presented as mean ± standard deviation (SD) or standard error (SE). The differences among all the groups were tested by analyzing the variances for the repeated measurement data based on whether the data was normally distributed (normal distribution: *t* test and ANOVA for continuous variables and χ^2^ test for categorical variable; non-normal distribution: Mann-Whitney U test and Kruskal-Wallis H test). Then the spearman correlations between the antibiotic exposure with anthropometric parameters and compositions of gut microbiota were determined by the multiple covariate-adjusted regressions by controlling the maternal age, BMI before delivery, gestational age, mode of delivery, sex, antibiotic types, usage and related disease treatment.

## Results

### Basic characteristics of the mother-infant pairs in this cohort

**Fig. 1:**
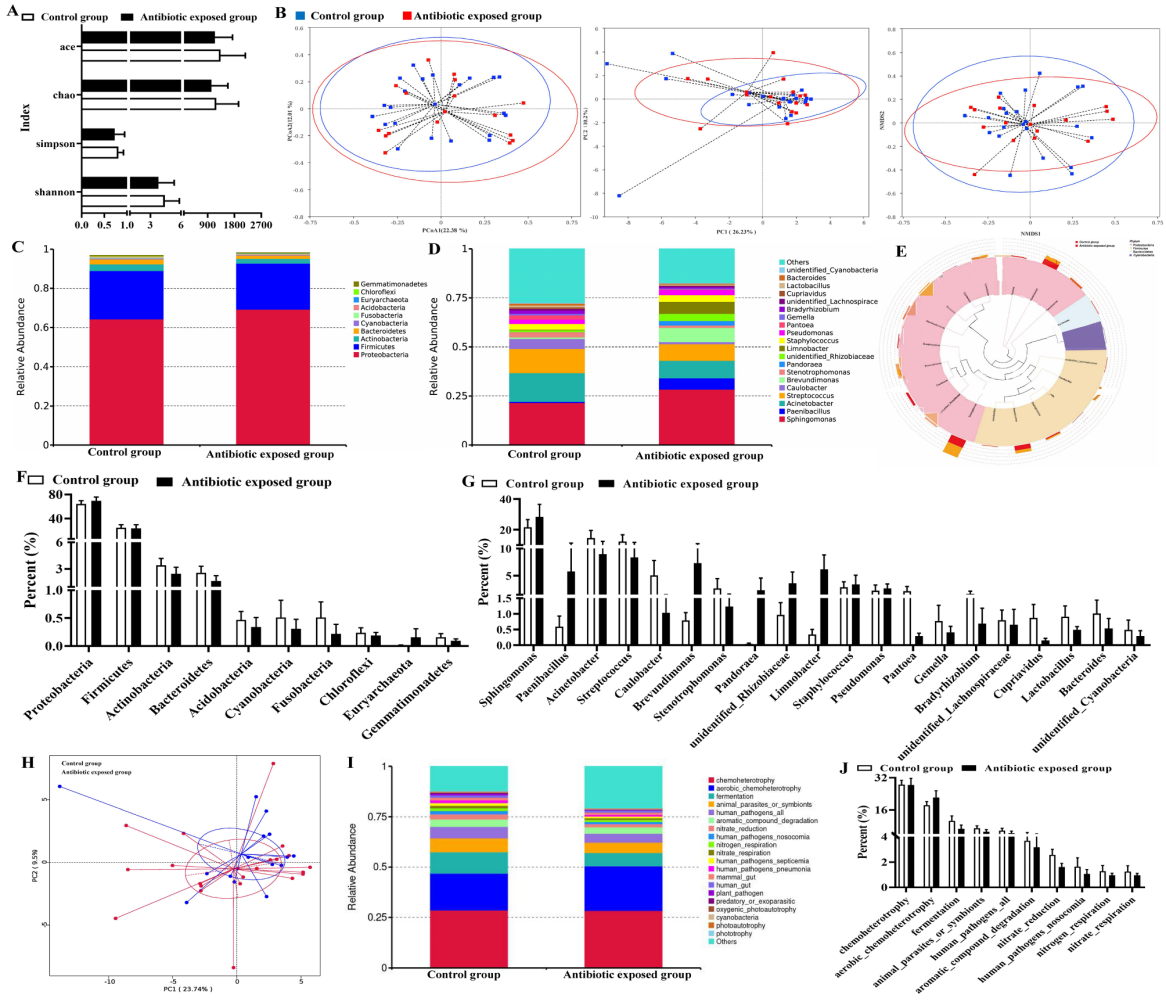
Diversity, compositions and functional features of milk microbiota between the control and antibiotic exposed groups. A: α diversity index. B: β diversity index. C and F: Top ten most abundant phyla. D and G: Relative twenty most abundant genera. E: Ternary-plot analysis from the phylum to genus levels. H: PCA analysis of functional features. I and J: Relative top twenty and ten most abundance of functional features

The characteristics of mother-infant pairs was presented as Table 1; Fig. 1, in which the majority of maternal age was between 25 and 35 years old (85.46%). They had normal pre-pregnancy BMI without smoking and drinking history, pre-existing hypertension and preeclampsia. Meanwhile, their infants (52.20% boys and 66.03% natural birth) were all singleton birth with normal growth and development. They were more than 6 months breastfed (68.46%) and introduced to the solid foods after 6 months (69.02%). Futhermore, there were no significant differences of all these maternal and infant characteristics at the baseline, and compositions of milk microbiota between the control and antibiotic exposed groups (*P* > 0.05).

**Table 1:** Descriptive characteristics of the subjects in different groups

Index*	Control group (n = 1005)	Antibiotic exposed group (n = 1135)	*P*
**Maternal Characteristics** Age at childbirth(years)	30.73 ± 3.51	29.89 ± 3.42	0.668
Height (cm)	162.97 ± 4.77	163.14 ± 5.09	0.714
Pre-pregnancy weight (kg)	57.07 ± 8.88	57.25 ± 8.24	0.344
Pre-pregnancy BMI (kg/m^2^)	21.49 ± 3.28	21.51 ± 3.09	0.985
Pre-pregnancy overweight (Yes, %)	182 (18.11)	204 (17.97)	0.955
Pre-pregnancy obesity (Yes, %)	31 (3.08)	34 (3.00)	0.900
Prenatal weight (kg)	71.16 ± 3.17	72.03 ± 4.19	0.854
Prenatal BMI (kg/m^2^)	26.79 ± 5.04	27.06 ± 2.36	0.652
Weight gain (kg)	14.09 ± 4.43	14.78 ± 4.36	0.688
BMI gain (kg/m^2^)	5.31 ± 1.34	5.55 ± 1.05	0.408
Education (n, %) Bachelor degree or above	753 (74.92)	788 (69.43)	0.073
High school	203 (20.20)	318 (28.02)	
Middle school and below	49 (4.88)	29 (2.55)	
Medical History during the pregnancy (Yes, %) Hypertension	41 (4.08)	34 (3.00)	0.195
Diabetes	27 (2.69)	35 (3.08)	0.608
Hypothyroidism	17 (1.69)	21 (1.85)	0.870
Anemia	305 (30.35)	309 (27.23)	0.114
Respiratory infection	91 (9.05)	114 (10.04)	0.462
Digestive system diseases	12 (1.19)	16 (1.41)	0.707
Allergic diseases	30 (2.99)	27 (2.38)	0.421
Antibiotic exposure during the pregnancy (Yes, %)	144 (14.33)	171 (15.07)	0.669
**Infant Characteristics** Sex (Boy, %)	532 (52.94)	585 (51.54)	0.647
Gestational age (Week)	39.18 ± 1.13	39.23 ± 1.08	0.612
Mode of delivery (Natural birth, %)	672 (66.87)	741 (65.29)	0.708
Breast-feeding duration (n, %) Less than 3 month	38 (3.78)	47 (4.14)	0.842
3 to 6 months	276 (27.46)	314 (27.67)	
6 to 12 months	486 (48.36)	532 (46.87)	
≥ 12 months	205 (20.40)	242 (21.32)	
Introduction to solid foods (n, %) <6 month	287 (28.56)	376 (33.13)	0.052
≥ 6 month	718 (71.44)	759 (66.87)	

Note: * The qualitative data was shown as n (%) and analyzed by χ^2^ test between the control and antibiotic exposed groups, while the other quantitative data was shown as mean ± standard deviation (SD) and analyzed by *t* test.

### Antibiotic exposure of the participants in the first one year of life

**Fig. 2:**
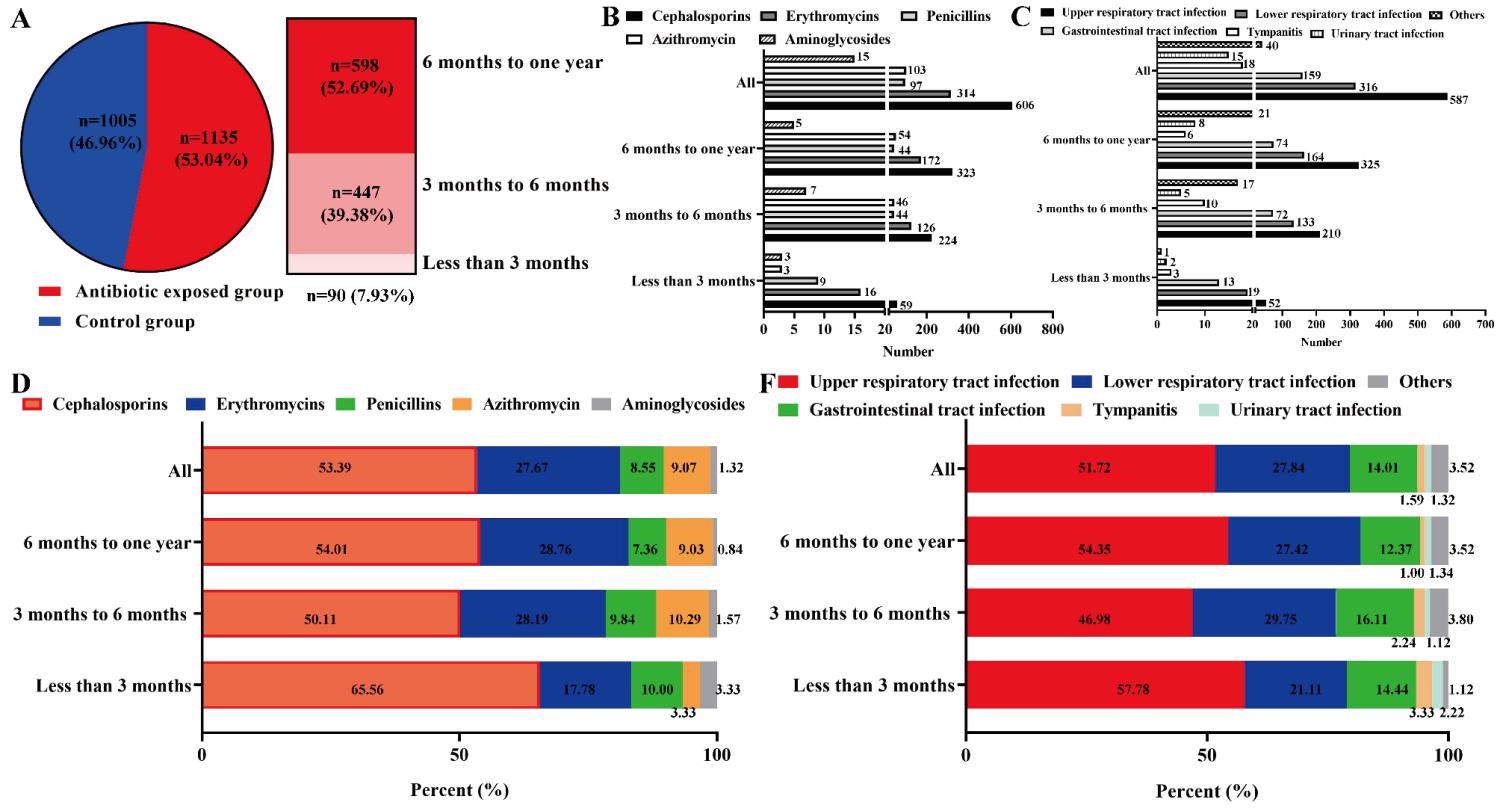
Characteristics of antibiotic exposure among the infants at different ages in the first one year of life. A: Antibiotic exposure at different ages during the first one year of life. B and D: Numbers and percents of different types for antibiotic exposure respectively. C and F: Numbers and percents of different diseases for antibiotic exposure

As shown in Table 1; Figs. 2, **53**.04% infants (n = 1135) were dispensed at least one course of antibiotic exposure within the first one year of life (Fig. 2 A), in which there were respectively 7.93% (90/1135), 39.38% (447/1135) and 52.69% (598/1135) infants who were firstly given antibiotic exposure at less than 3 months, 3 to 6 months and 6 months to one year. Notably,, the top five most important types of antibiotics were Cephalosporins (53.39%), Erythromycins (27.67%), Penicillins(8.55%), Azithromycin (9.07%) and Aminoglycosides (1.32%) (Fig. 2B and D), which were most commonly used for the upper respiratory tract infection (51.72%), lower respiratory tract infection (27.84%), gastrointestinal tract infection (14.01%), tympanitis (1.59%) and urinary tract infection (1.32%) (Fig. 2 C and 2 F). Moreover, there were not significant differences of the above antibiotic types and disease treatment among the diffierent age subgroups (*P* > 0.05).

### Antibiotic exposure in the first one year of life increases the risk of childhood overweight and obesity from one year to two and a half years

**Fig. 3:**
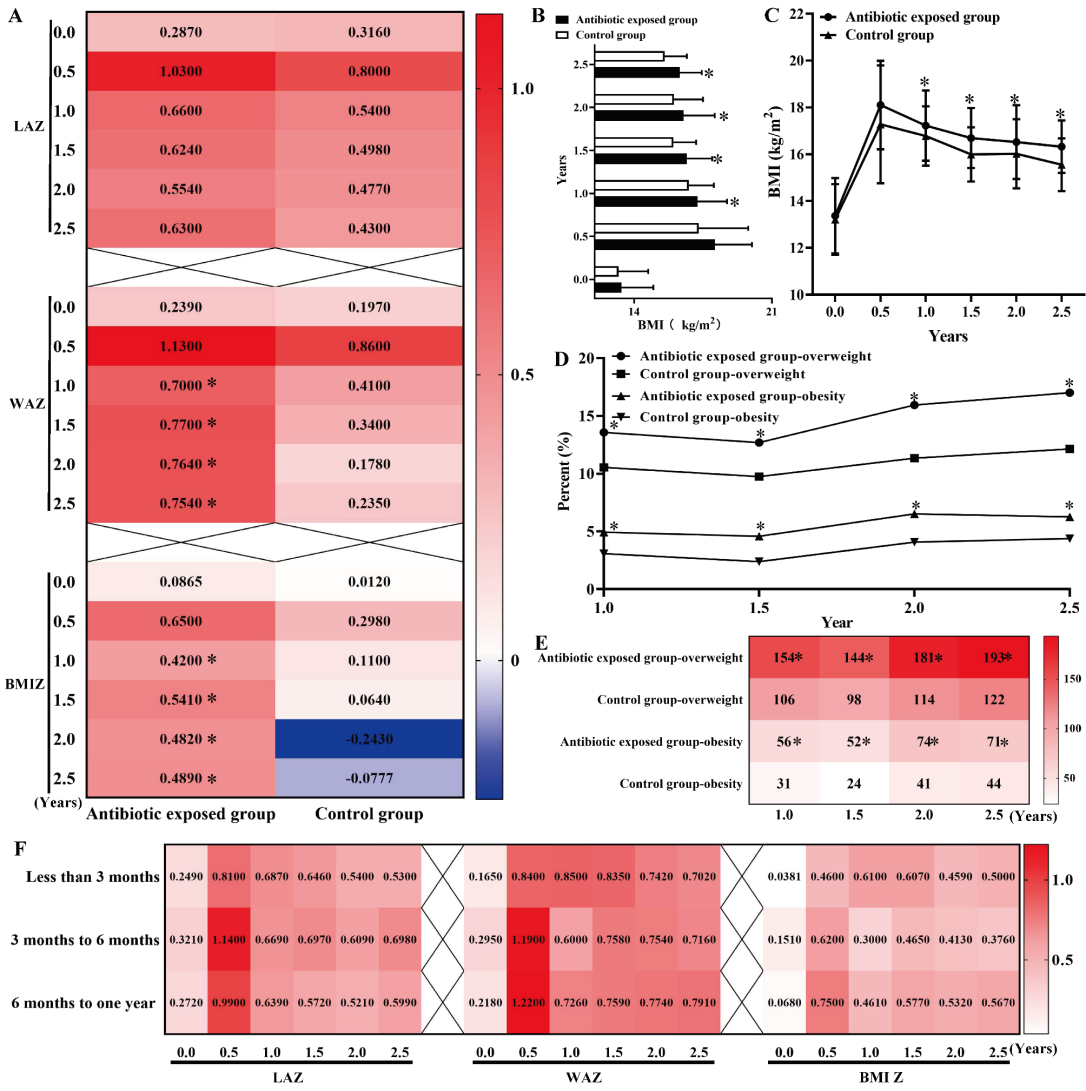
Antibiotic exposure in the first one year of life increases the risk of childhood overweight and obesity from one year to two and half years. A: The values of LAZ, WAZ and BMI Z. B and C: BMI at different ages. D and E: The prevalence and numbers of childhood overweight and obesity. F: LAZ, WAZ and BMI Z in the different antibiotic exposure subgroups. Note: BMI: body mass index, WAZ: weight for age Z score, LAZ: length for age Z score, BMI Z: BMI for age Z score. *Compared to the control group, *P* < 0.05

Comparing with the control group, antibiotic exposure during the first one year of life was associated with the increasing prevalence of childhood overweight and obesity from one year to two and a half years (Table 2; Fig. 3D, E and P < 0.05), with the significantly higher BMI (Table 2; Fig. 3B C), WAZ (Fig. 3 A) and BMI Z (Fig. 3 A) (*P* < 0.05). Among the subgroups from less than 3 months, 3 months to 6 months and 6 months to one year, there were no significant differences of height, weight, BMI, LAZ, WAZ, BMI Z and prevalences of childhood overweight and obesity from birth to two and a half years (*P* > 0.05, Table 3; Fig. 3 F).

**Table 2:** Effects of antibiotic exposure in the first one year of life on the growth and development outcomes from birth to two and a half years old

Variables*	Control group (n = 1005)	Antibiotic exposed group (n = 1135)	*t/*χ^2^	*P*
At birth Height (cm)	50.09 ± 1.71	50.08 ± 1.69	0.142	0.887
Weight (kg)	3.32 ± 0.44	3.38 ± 0.47	1.164	0.246
BMI (kg/m^2^)	13.23 ± 1.52	13.47 ± 1.61	0.611	0.541
Six months Height (cm)	68.57 ± 2.56	68.99 ± 2.85	1.209	0.228
Weight (kg)	8.25 ± 1.38	8.64 ± 1.21	1.624	0.106
BMI (kg/m^2^)	17.54 ± 2.52	18.15 ± 1.89	1.658	0.099
One year Height (cm)	76.32 ± 2.58	76.54 ± 2.84	1.192	0.234
Weight (kg)	9.79 ± 1.06	10.11 ± 1.24	1.201	0.230
**BMI (kg/m** ^**2**^ **)**	**16.78 ± 1.27**	**17.23 ± 1.50**	**4.547**	**< 0.001**
**Overweight (n, %)**	**106 (10.55)**	**154 (13.57)**	**4.558**	**0.033**
**Obesity (n, %)**	**31 (3.08)**	**56 (4.93)**	**4.674**	**0.031**
One and a half years Height (cm)	82.89 ± 2.81	83.13 ± 2.86	1.437	0.151
**Weight (kg)**	**11.00 ± 1.12**	**11.61 ± 1.27**	**2.328**	**0.020**
**BMI (kg/m** ^**2**^ **)**	**16.01 ± 1.15**	**16.80 ± 1.28**	**10.718**	**< 0.001**
**Overweight (n, %)**	**98 (9.75)**	**144 (12.69)**	**4.581**	**0.032**
**Obesity (n, %)**	**24 (2.39)**	**52 (4.58)**	**7.487**	**0.006**
Two years Height (cm)	88.26 ± 3.15	88.09 ± 3.08	0.962	0.338
Weight (kg)	12.56 ± 1.38	12.74 ± 1.45	1.495	0.135
**BMI (kg/m** ^**2**^ **)**	**16.12 ± 1.48**	**16.52 ± 1.58**	**2.868**	**0.004**
**Overweight (n, %)**	**114 (11.34)**	**181 (15.95)**	**9.506**	**0.002**
**Obesity (n, %)**	**41 (4.08)**	**74 (6.52)**	**6.242**	**0.012**
Two and a half years Height (cm)	92.77 ± 3.40	93.53 ± 3.33	1.454	0.147
**Weight (kg)**	**13.40 ± 1.42**	**14.31 ± 1.54**	**7.547**	**< 0.001**
**BMI (kg/m** ^**2**^ **)**	**15.57 ± 1.13**	**16.32 ± 1.12**	**8.507**	**< 0.001**
**Overweight (n, %)**	**122 (12.14)**	**193 (17.01)**	**10.050**	**0.002**
**Obesity (n, %)**	**44 (4.38)**	**71 (6.26)**	**4.130**	**0.043**

Note: * The prevalence of overweight and obesity was shown as n (%) and analyzed by χ^2^ test between the control and antibiotic exposed groups, while the other variables were shown as mean ± standard deviation (SD) and analyzed by *t* test, in which the data with statistically significant differences were shown in bold.

**Table 3:** Effects of antibiotic exposure in different stages during the first one year of life on the growth outcomes from birth to two and a half years old

Variables*	Less than 3 months (n = 90)	3 to 6 months (n = 447)	6 months to one year (n = 598)	*F/x* ^*2*^	*P*
At birth Height (cm)	50.00 ± 1.83	50.14 ± 1.78	50.06 ± 1.61	0.238	0.789
Weight (kg)	3.34 ± 0.54	3.41 ± 0.50	3.37 ± 0.44	0.780	0.459
BMI (kg/m^2^)	13.31 ± 1.52	13.55 ± 1.79	13.45 ± 1.55	0.780	0.459
Six months Height (cm)	68.59 ± 2.76	69.29 ± 3.13	68.79 ± 2.54	0.864	0.423
Weight (kg)	8.40 ± 1.06	8.69 ± 1.25	8.66 ± 1.19	0.637	0.530
BMI (kg/m^2^)	17.82 ± 1.87	18.08 ± 1.98	18.25 ± 1.79	0.509	0.602
One year Height (cm)	76.64 ± 2.80	76.65 ± 3.02	76.43 ± 2.75	0.306	0.736
Weight (kg)	10.31 ± 1.29	10.03 ± 1.10	10.11 ± 1.31	1.053	0.350
BMI (kg/m^2^)	17.51 ± 1.53	17.06 ± 1.34	17.26 ± 1.60	2.030	0.133
Overweight (n, %)	10 (11.11)	63 (14.09)	81 (13.55)	0.569	0.752
Obesity (n, %)	5 (5.56)	18 (4.03)	33 (5.52)	1.294	0.524
One and a half years Height (cm)	83.17 ± 2.91	83.31 ± 2.92	83.00 ± 2.81	0.841	0.432
Weight (kg)	11.69 ± 1.50	11.59 ± 1.29	11.61 ± 1.21	0.165	0.848
BMI (kg/m^2^)	16.79 ± 1.61	16.58 ± 1.19	16.75 ± 1.26	1.492	0.226
Overweight (n, %)	11 (12.23)	58 (12.98)	75 (12.54)	0.062	0.969
Obesity (n, %)	4 (4.44)	21 (4.70)	27 (4.52)	0.024	0.988
Two years Height (cm)	87.99 ± 3.12	88.27 ± 3.14	88.01 ± 3.08	0.559	0.572
Weight (kg)	12.68 ± 1.71	12.72 ± 1.54	12.76 ± 1.34	0.106	0.899
BMI (kg/m^2^)	16.47 ± 1.58	16.42 ± 1.27	16.59 ± 1.22	1.235	0.292
Overweight (n, %)	15 (16.67)	72 (16.11)	94 (15.72)	0.067	0.967
Obesity (n, %)	6 (6.67)	32 (7.16)	36 (6.02)	0.548	0.760
Two and a half years Height (cm)	93.14 ± 3.51	93.77 ± 3.35	93.42 ± 3.29	0.552	0.576
Weight (kg)	14.19 ± 1.71	14.25 ± 1.52	14.37 ± 1.53	0.279	0.757
BMI (kg/m^2^)	16.33 ± 1.39	16.17 ± 1.06	16.43 ± 1.09	1.698	0.185
Overweight (n, %)	16 (17.78)	76 (17.01)	101 (16.89)	0.044	0.878
Obesity (n, %)	4 (4.45)	28 (6.26)	39 (6.52)	0.576	0.750

Note: * The prevalence of overweight and obesity was shown as n (%) and analyzed by χ^2^ test among the less than 3 months, 3 months to 6 months and 6 months to one year groups, while the other variables were shown as mean ± standard deviation (SD) and analyzed by the analysis of variance (ANOVA).

### Effects of antibiotic exposure in the first one year of life on the diversity and compositions of gut microbiota at two and a half years

**Fig. 4:**
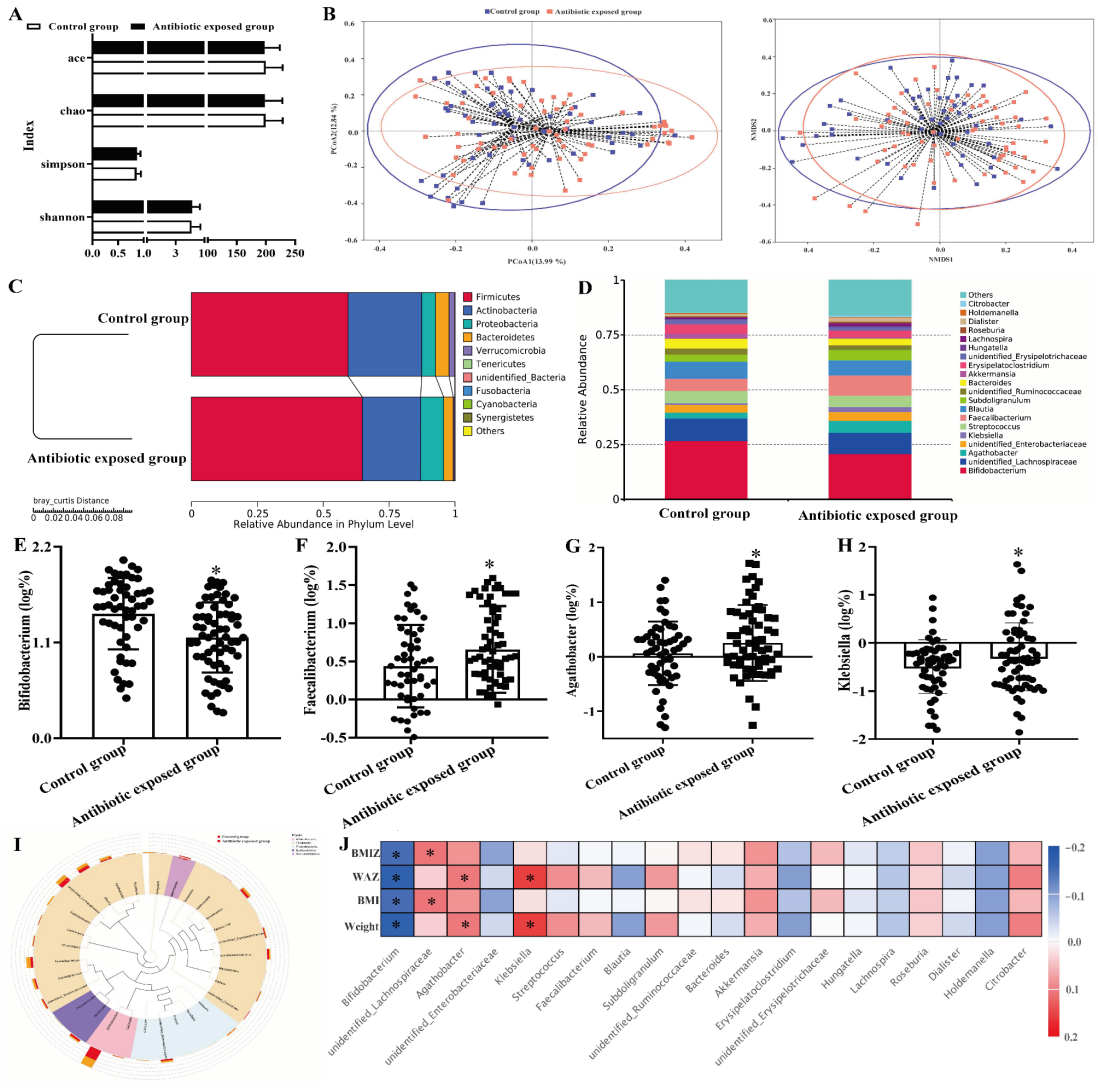
Effects of antibiotic exposure in the first one year of life on the diversity and compositions of gut microbiota at the two and a half years. A: α diversity index. B: β diversity index. C: Relative ten most abundant phyla. D: Relative twenty most abundant genera. E to H: The percents of *Bifidobacterium*, *Faecalibacterium*, *Agathobacter* and *Klebsiella*. I: Ternary-plot analysis from the phylum to genus levels. J: Spearman correlations between twenty most abundant genera with the growth and development related indicators (weight, BMI, WAZ and BMI Z). *Compared to the control group, *P* < 0.05

To assess the effects of early-life antibiotic exposure on the bacterial diversity, 60 subjects were randomly selected to measure the richness, phylogenetic diversity, and evenness of gut microbiota in the control (n = 1005) and antibiotic exposed groups (n = 1135). Comparing with the control group, the α-diversity indicators were not significantly suppressed among the individuals by the antibiotic administration (Fig. 4 A, P > 0.05). Moreover, β-diversity, measuring the similarities as the composition and recovery of an entire microbial ecosystem, was also not significantly different between the control and antibiotic exposed groups (Fig. 4B, P > 0.05).

The communities of gut microbiota were followed by a predictable pattern throughout the two and a half years in Fig. 4; Table 4. To structurally organize the compositions of gut microbiota, no significant differences were shown between the control and antibiotic exposed groups at the phylum level (Fig. 4 C and Table 4, *P* > 0.05). However, the percents of *Faecalibacterium* (Fig. 4 F), *Agathobacter* (Fig. 4G) and *Klebsiella* (Fig. 4 H, P < 0.05) were higher, with the decreasing percentage of *Bifidobacterium* (Fig. 4E, P < 0.05) in the antibiotic exposed group than those in the control group at the genus level (Fig. 4D and I).

**Table 4:** Effects of antibiotic exposure during the first one year of life on the compositions of gut microbiota at the two and a half years

Gut microbiota (%)*	Control group (n = 60)	Antibiotic exposed group (n = 60)	*t/Z*	*P*
Firmicutes	59.39 ± 2.34	64.85 ± 2.49	1.597	0.113
unidentified_Lachnospiraceae	10.25 ± 1.35	9.56 ± 1.15	0.391	0.697
**Faecalibacterium**	**5.57 ± 0.98**	**9.20 ± 1.31**	**2.226**	**0.028**
Blautia	7.79 ± 0.89	6.89 ± 0.61	0.862	0.390
**Agathobacter**	**2.65 ± 0.61**	**5.51 ± 1.27**	**2.028**	**0.046**
Streptococcus^&^	5.65 ± 1.18	5.22 ± 0.87	0.301	0.765
Subdoligranulum-i	3.23 ± 0.56	4.77 ± 0.87	1.493	0.138
Erysipelatoclostridium	4.41 ± 0.80	3.06 ± 0.48	1.449	0.151
unidentified_Ruminococcaceae	2.73 ± 0.64	2.07 ± 0.38	0.917	0.361
Fusicatenibacter^&^	1.58 ± 0.24	1.74 ± 0.29	0.398	0.691
Dialister	1.05 ± 0.41	1.73 ± 0.54	0.976	0.331
Anaerostipes	1.22 ± 0.19	1.69 ± 0.33	1.259	0.211
unidentified_Erysipelotrichaceae	2.18 ± 0.64	1.60 ± 0.28	0.831	0.409
Lachnospira	0.71 ± 0.27	1.25 ± 0.44	1.004	0.318
Holdemanella	0.51 ± 0.38	0.35 ± 0.21	0.361	0.719
Actinobacteria^&^	27.93 ± 2.63	22.13 ± 2.29	1.666	0.099
**Bifidobacterium** ^&^	**26.79 ± 2.57**	**20.87 ± 2.13**	**2.032**	**0.045**
Proteobacteria	5.25 ± 0.84	8.71 ± 1.85	1.705	0.092
unidentified_Enterobacteriaceae	3.52 ± 0.70	3.94 ± 0.93	0.351	0.726
**Klebsiella**	**0.72 ± 0.19**	**2.37 ± 0.84**	**2.016**	**0.050**
Citrobacter	0.23 ± 0.054	0.77 ± 0.32	1.626	0.109
Bacteroidetes	5.17 ± 1.05	3.63 ± 0.66	1.284	0.202
Bacteroides	4.57 ± 0.95	3.13 ± 0.62	1.262	0.210
Verrucomicrobia	2.20 ± 0.81	0.62 ± 0.34	1.814	0.074
Akkermansia^&^	2.20 ± 0.81	0.62 ± 0.34	1.813	0.074
Tenericutes	0.011 ± 0.0067	0.017 ± 0.0091	0.489	0.626
unidentified_Bacteria	0.021 ± 0.0041	0.015 ± 0.0033	1.301	0.196
Fusobacteria^&^	0.0071 ± 0.0019	0.0091 ± 0.0029	0.549	0.584
Cyanobacteria	0.0069 ± 0.0025	0.0044 ± 0.0012	0.950	0.344
Synergistetes	0.00086 ± 0.00060	0.0013 ± 0.00074	0.414	0.680
Others	0.0091 ± 0.0017	0.011 ± 0.0037	0.447	0.656

Note: *The compositions of gut microbiota at the phylum and genus levels were shown as mean ± standard error (SE), in which Mann-Whitney U test was used to evaluate the percentages of gut microbiota (&) under the non-normal distribution, while the other variables were analyzed by *t* test, in which the data with statistically significant differences were shown in bold.

### Associations between the antibiotic exposure in the first one year of life with childhood overweight/obesity and significant compositions of gut microbiota at the two and a half years

All these above significant results were then confirmed using the covariate-adjusted analyses by controlling the maternal age, BMI before delivery, gestational age, mode of delivery, sex, antibiotic types, usage and related disease treatment (Table 5), in which there were positively independent associations between the antibiotic exposure in the first one year of life with childhood overweight and obesity from one year to two and a half years. Furthermore, positive associations were apparent between the antibiotic exposure and related anthropometric parameters (weight, BMI, WAZ and BMI Z) at age one year, one and a half years, two years and two and a half years, which were not significantly shown with the values of LAZ.

**Table 5:** Adjusted associations between antibiotic exposure with growth outcomes and related Z scores

Variables	Estimate (95% CI)	*P*
**Outcomes at one year** Overweight (RR)	2.164 (1.528, 3.063)	< 0.001
Obesity (RR)	2.932 (1.370, 6.278)	0.006
Weight (kg) (β)	-0.075 (-0.237, 0.087)	0.365
BMI (kg/m^2^) (β)	0.450 (0.256, 0.644)	< 0.001
LAZ (β)	0.118 (-0.030, 0.267)	0.117
WAZ (β)	0.293 (0.162, 0.423)	< 0.001
BMI Z (β)	0.319 (0.186, 0.452)	< 0.001
**Outcomes at one and a half years** Overweight (RR)	2.245 (0.832, 6.063)	0.110
Obesity (RR)	3.985 (1.900, 8.357)	< 0.001
Weight (kg) (β)	0.615 (0.487, 0.743)	< 0.001
BMI (kg/m^2^) (β)	0.710 (0.580, 0.840)	< 0.001
LAZ (β)	0.570 (-0.310, 1.452)	0.202
WAZ (β)	0.430 (0.339, 0.522)	< 0.001
BMI Z (β)	0.477 (0.389, 0.565)	< 0.001
**Outcomes at two years** Overweight (RR)	4.594 (3.275, 6.443)	< 0.001
Obesity (RR)	4.507 (1.970, 10.312)	< 0.001
Weight (kg) (β)	0.868 (0.713, 1.022)	< 0.001
BMI (kg/m^2^) (β)	0.971 (0.831, 1.111)	< 0.001
LAZ (β)	0.078 (-0.029, 0.184)	0.152
WAZ (β)	0.571 (0.467, 0.676)	< 0.001
BMI Z (β)	0.705 (0.602, 0.808)	< 0.001
**Outcomes at two and a half years** Overweight (RR)	2.936 (1.905, 4.527)	< 0.001
Obesity (RR)	3.073 (2.300, 4.107)	< 0.001
Weight (kg) (β)	0.907 (0.671, 1.143)	< 0.001
BMI (kg/m^2^) (β)	0.777 (0.597, 0.956)	< 0.001
LAZ (β)	0.206 (-0.041,0.453)	0.058
WAZ (β)	0.519 (0.382, 0.656)	< 0.001
BMI Z (β)	0.566 (0.433, 0.700)	< 0.001

The associations between antibiotic exposure with significant compositions of gut microbiota at two and a half years were presented in Fig. 4 J, the percent of *Bifidobacterium* was negatively correlated with the variables of weight, BMI, WAZ and BMI Z at the two and a half years (*P* < 0.05), while the positive correlations were significantly obtained between the percents of *Agathobacter* and *Klebsiella* with weight and WAZ, which were also demonstrated between the percentage of *unidentified_ Lachnospiraceae* with BMI and BMI Z (*P* < 0.05).

## Discussion

With the increasing abuse of antibiotic exposure in the infancy, we are not only faced with the threat of antibiotic resistance, but also a rising concern about the potential long-lasting effects on the health, in which the occurrence and progression of obesity is a condition resulting from the complex interactions of genetic, dietary and lifestyle factors. So far, there are numerous elements that have been convincingly found to be associated with the implications of childhood overweight and obesity as reported in the previously systematic reviews, especially the higher rates of antibiotic prescriptions, and accelerated infant weight gain [33, 34]. Moreover, there was also a multi-centre cohort study that reported a strong link between prenatal antibiotic exposure in the second-trimester and increased risk of childhood obesity [13]. Furthermore, the interests in the line of inquiry had resurfaced alongside with the existing meta analysis, in which there were positive associations between early-life antibiotic exposure and odds of later childhood obesity using the animal models, with the weaker association data available from human studies [35]. Conversely, in the subgroup analysis of antibiotic exposure period, the subjects aged 6 to 12 months had no significant increases in the risk for childhood overweight by Trasande et al. [36]. Meanwhile, Gerber et al. reported that there were no significant associations between early-life antibiotic exposure and weight gain among the children through age 7 years. However, Saari A and Azad MB did not find any significant correlations between the risk of childhood obesity and postnatal antibiotic exposure in contrast with the previous findings, which was also no evidence for the increased risks for the development of obesity among the boys as previously stated [28, 37]. The above inconsistent conclusions were due to the methodological differences to cause it difficult to make the direct comparisons likely, which might explain the discrepancy in our findings, such as the host genetics, maternal BMI, environmental factors, types of infection and details of antibiotic exposure (timing, number of courses, doses and class). So following the strict inclusion criteria, including the healthy mother at 20–45 years without smoking and drinking history, birth at the gestational weeks of 37–41, and their newborns with normal growth and development indexes and exclusive breastfeeding from birth to six months in concordance with several prior studies [23–28], our findings in this birth cohort indicated that comparing with the control group, the infants with antibiotic exposure in the first one-year of life (mainly Cephalosporins and Erythromycins for the treatment of respiratory tract infection) had higher risk of childhood overweight and obesity at the early time points of age from one year to two and a half years, which were not significantly different among the subgroups (less than 3 months, 3 to 6 months, and 6 months to one year).

The mechanisms by which early-life antibiotic exposure indirectly modulates the later childhood overweight and obesity are still unclear. However, there are a number of hypotheses that antibiotic exposure during the first year of life-a critical exposure period in the development of gut microbiota-may have the great impacts on the excessive growth of childhood BMI from the indigestible polysaccharides, and a reduction in the intestinal defense [38, 39]. Meanwhile, many epidemiological studies had proved that infant gut microbiota were undergone a gradual succession with the age-dependent spatterns, and a large degree of inter-individual variations were occurred during the first two and three years of life. And our previous researches also had proved that the colonization of gut microbiota was significantly matured into the adulthood mode, which was mainly regulated by their milk microbiota, dietary pattern and so on. Thus, we examined whether antibiotic exposures and other disturbances in the first year of life similarly altered the childhood microbiota maturation at two and a half years. Furthermore, previous studies had suggested that the compositions of gut microflora were associated with the later obesity. Meanwhile, early-life antibiotic exposure could cause the decreasing phylogenetic diversity, richness and abundance of Actinobacteria (especially *Bifidobacteria*), and increasing percentages of *Bacteroidetes* and *Proteobacteria* (*Enterobacteriaceae, Staphylococcus, Clostridium* and *Enterococcus*) to cause the proliferation of potentially pathogenic bacteria [40]. Given the modifiable mature of gut microflora, the large shifts in the infant diets and environmental factors could ample the opportunity for the improved antimicrobial use, with the increasing *Clostridiales* and decreased *Bacteroidacea* [24, 28, 35–37]. To our knowledge, many evidence from animal and human studies had supported the concept that the colonization and development of gut microbiota could affect the infant growth to increase the risk of obesity, in which Ridaura et al. showed that the co-housing recipient mice harboring either the lean or obesity gut microbiota from human donors could prevent the increases in the mouse adiposity, who received the obesity gut microbiota [41]. Meanwhile, the longitudinal studies had identified that early-life antibiotic usage had profound short- and long-term effects on the later diversity and compositions of gut microbiota. Moreover, much larger and growing number of studies implicated the causal roles on the perturbations of gut microbiota in the development of obesity [42], so it is critical on identifying the risks, which are associated with the emerging prescription trends. In our study, the results proved that there were increasing percents of *Faecalibacterium, Agathobacter* and *Klebsiella*, and decreasing percentages of *Bifidobacterium* at the genus level, which were consistent with the past studies [23, 37]. The previous researches proved that the treatment with variety of antibiotic exposure had been found to decrease the relative percentages of microbial taxa such as *Bifidobacteriaceae*, *Bacilli*, and *Lactobacillales* in ways that were predisposed the children to increase the weight gain by reducing the bacterial diversity, increasing the abundance of endotoxin producing organisms, and depleting the beneficial bacteria and organisms to play a key role on the metabolism [43]. There was another study examining the consistency of antibiotic perturbation on the significant reductions in both bacterial load and diversity, including depletion of *Bacteroidales* and the marked enrichment of *Lactobacillus* [44]. And the mice, who were exposed to the sub-therapeutic antibiotic exposure through drinking water, had the significant decreases in the percents of *Bacteroides* to *Firmicutes* [45].

There were still many limitations in this birth cohort. Firstly, the major limitation was that the early-life antibiotic exposure in the first one year of life was obtained by the retrospective non-randomized data collection using the questionnaires, so it was unable to determine the causative correlations between early-life antibiotic exposure and childhood obesity by the prospective randomized clinical studies. The other limitation was included a lack of determined information on the dietary structure to avoid the excessive energy intake to cause the childhood overweight and obesity. Furthermore, the sample size was small, so we were able to conduct further analyses on the correlations between early-life antibiotic exposure and childhood overweight/obesity to better account for the confounding factors with much larger cohort studies.

## Conclusion

In summary, our results from this birth cohort proved that the antibiotic exposure during the first one year of life was 53.04% among the infants. They were mainly Cephalosporins and Erythromycins for the treatment of respiratory tract infection, without significant differences among the different age subgroups. What is more, there were positively potential associations between early-life antibiotic exposure with the accelerated childhood overweight and obesity from one year to two and a half years, which might be regulated by the abnormal development of gut microbiota, with the disorders of *Faecalibacterium, Agathobacter, Klebsiella* and *Bifidobacterium* at the genus level. Therefore, the implementation of this project could propose the theoretical basis for rationalizing the personalized antibiotic exposure among the infants to truly reflect the fairness of public health. It will not only ensure the reasonable utilization of early-life antibiotic exposure in the first one year of life, but also provide a new idea for the prevention of later childhood overweight and obesity.

## Electronic supplementary material

Below is the link to the electronic supplementary material.

Supplementary Material 1

## Data Availability

The data and materials that support the findings of this study are available from the corresponding author upon the reasonable requests.

## Abbreviations

BMI: body mass index.
LAZ: length for age Z score.
WAZ: weight for age Z score.
BMIZ: BMI for Z score.
PCA: Principal coordinate analysis.
NMDS: Non-metric Multi-Dimensional Scaling analysis.
PICRUSt: Phylogenetic Investigation of Communities by Reconstruction of Unobserved States.
SD: standard deviation.
SE: standard error.
