# Supplementary figures and images for: Early-life antibiotic exposure increases the risk of childhood overweight and obesity in relation to dysbiosis of gut microbiota: a birth cohort study
Source: Ann Clin Microbiol Antimicrob. 2022 Nov 3;21:46. doi: 10.1186/s12941-022-00535-1 (PMC9635112; doi:10.1186/s12941-022-00535-1)

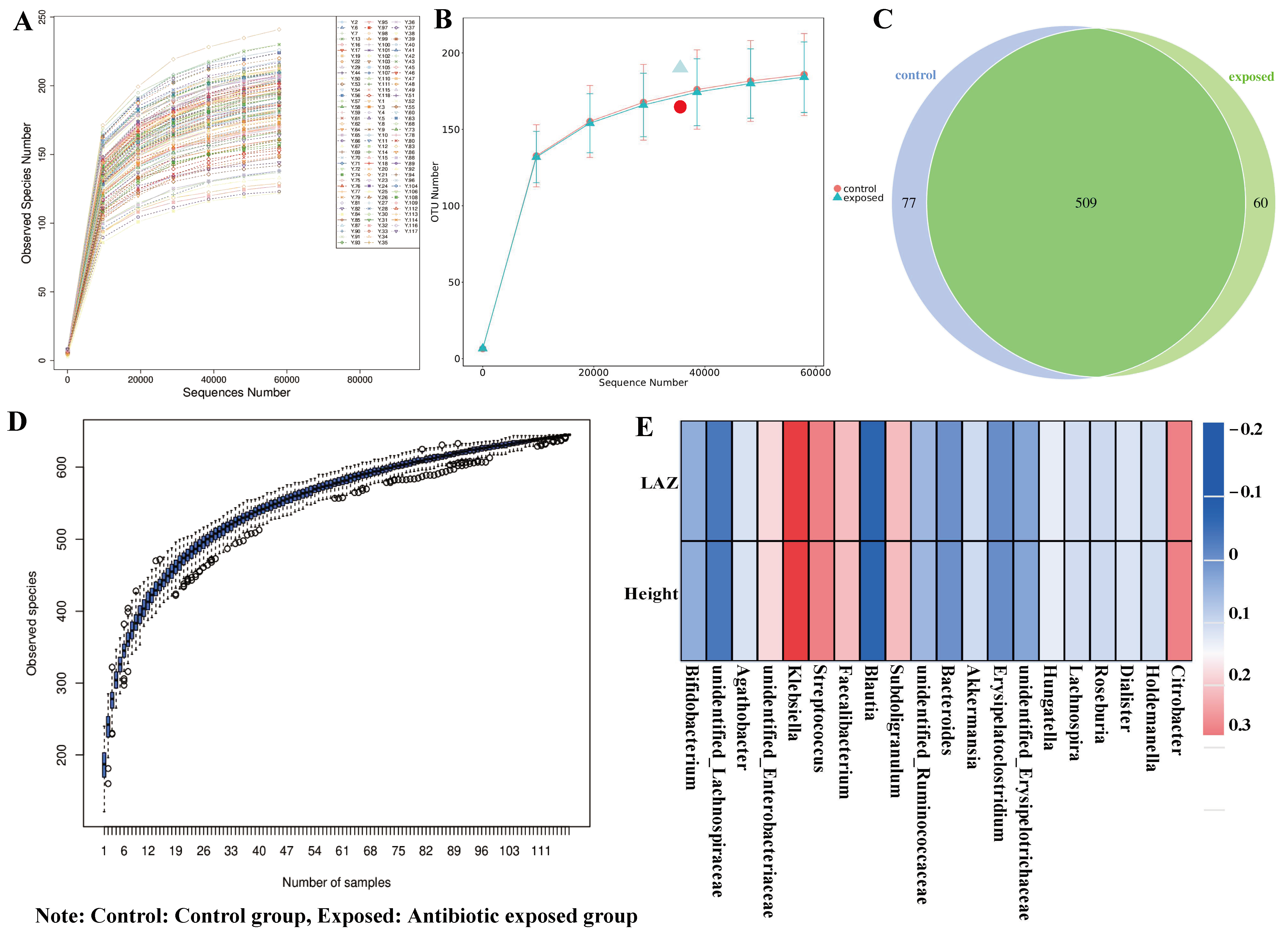

Supplement: Supplementary file 1 — Supplementary Material 1 [file 12941_2022_535_MOESM1_ESM.tiff]
